# Supplementary material for: Equipping computational pathology systems with artifact processing pipelines: a showcase for computation and performance trade-offs
Source: BMC Med Inform Decis Mak. 2024 Oct 7;24:288. doi: 10.1186/s12911-024-02676-z (PMC11457387; doi:10.1186/s12911-024-02676-z)
Supplement: Supplementary file 1 — Supplementary Material 1: Peer review [file 12911_2024_2676_MOESM1_ESM.zip › peer review/bf1cc57d-2d0a-4df4-b824-a51d578b6a75 - response letter v2.pdf]

Equipping Computational Pathology Systems with Artifact Processing Pipelines: A Showcase for Computation and Performance Trade-offs

Authors: Neel Kanwal, Farbod Khoraminia, Umay Kiraz, Andrés Mosquera-Zamudio, Emiel A.M. Janssen, Carlos Monteagudo, Tahlita C.M. Zuiverloon, Chunming Rong, Kjersti Engan

Second Revision: Detailed Response to the Reviewers

July 24, 2024

We thank the reviewers for spending time in this round. However, we are not clear whether Reviewer#3 and Reviewer#4 are exactly same reviewers from previous round. From comments, it sounds that Reviewer#4 is probably one of the same person from previous round. However, Reviewer#3's comments does not align with revised copy of the manuscript and we have our deep concern with technical and factual grounds of the feedback.

To facilitate reading this document, we have highlighted the feedback and concerns in red, our replies in black, and text additions in blue.

Reviewer 3

This paper proposes a DL approach for detecting artifacts in WSIs to improve the reliability of computational pathology systems. My major concerns are listed as follows:
1. The paper does not compare the proposed approach with existing state-of-the-art methods for artifact detection. The authors should provide a comprehensive comparison with relevant methods from the literature. In the last few years, several methods (most of them open-source) to evaluate artefact in digital pathology (es. HistoQC);

We have mentioned in the related work section that artifact detection is a largely ignored area, and there are only a handful of papers proposing detailed artifact detection methods for histopathology. However, the paper provides a comparison with the state-of-the-works from the literature in Table 3 (on page 19),

added in the first revision.

Table 3: **Comparison of the proposed mixture of experts (MoE) against the literature on identical classification tasks.** Note that the reported methods were developed using different data under different experimental setups. Thus, the results are provided for reference, not as a direct comparison. The best results are marked in bold, and the second-best results are underlined.

Task and method from literature	Accuracy (%)
Folded tissue detection by [85]	92.17
Folded tissue detection by [76]	96.7
Blur detection by [86]	93.2
Blur detection by [71]	94.4
Air bubble detection by [85]	87.33
Air bubble detection by [87]	91.5
Blood detection by [69]	85.0
Damaged tissue detection by [88]	90.0
Five artifacts - MoE-DCNNs (Ours)	<u>97.82</u>
Five artifacts - MoE-ViTs (Ours)	**97.84**

Moreover, HistoQC, as the reviewer mentions, is mainly for outlier detection in a cohort of WSIs, not for removing artifacts areas within a WSI patch-based manner, as our approach does. Unsurprisingly, the HistoQC paper do not report accuracy or sensitivity metrics. The following sentence is added in the related work.

HistoQC was proposed to perform a content-based evaluation for detecting outliers in a cohort of WSIs using a combination of image metrics and supervised classifiers. However, it did not remove or detect each artifact in smaller regions on WSIs.

2. While the authors create a pipeline that provides quantitative data, there is no numerical control or validation of the errors. For example, the authors should provide an absolute error comparison between manually and automatically estimated artifact areas to demonstrate the accuracy of their approach.

We are not sure we understand what the reviewer means here as quantitative data and numerical control. The authors have provided a quantitative evaluation (Table 6 on page 23), which compares manually annotated artifacts (by experts) and what our algorithm detects as histological artifacts. In addition, we conducted a qualitative and quantitative evaluation of six WSIs, where we performed external out-of-distribution evaluation by three pathologists to capture the usefulness of the proposed method. Interestingly, no other literature

validates their proposed method to this extent.

The following text is added in the first paragraph on page 22 to address the comment and make a better connection of quantitative evaluation with mathematical equations discussed in the methodology.

At the inference level, every patch from the WSI tissue foreground is used to perform predictions. Therefore, the error is calculated using evaluation metrics reported in Section 4.6, where the ground truth label of a patch is only an artifact if it overlaps 70% with artifact annotation as described in Section 4.2.

3. The paper lacks a discussion on important practical considerations, such as computation times, compatibility with different formats and scanners, and the effect of magnification on the proposed approach.

The paper does mention the computational complexity and the time it takes the models to run over a single patch. These numbers can be seen in Table 5 on page 21 and discussed in the text where Table 5 is cited (on page 20). Additionally, the external out-of-distribution ($s_1 - s_6$) whole slide images are produced by different scanners, formats, and tissue types. Please find the results in Table 6. All experiments are done at the full magnification level (40x), this is stated in the paper on page 8. We want to state that many papers in computational pathology only look at one magnification level.

4. The computational time and resources required for training and inference are not reported. This information is essential for readers to assess the practicality and feasibility of the proposed approach, especially when considering the trade-offs between performance and computational complexity.

Subsection 4.7 on page 18 does mention the implementation details and experimental setup required for training and inference. Table 5 describes the throughput to understand the computational time needed for inference per patch to run the proposed method. As WSIs can be of different sizes, we considered reporting throughput as a more informative metric, as it scales linearly with the number of patches. Also, we have provided a very detailed discussion of the results in the paper, which can help the reader understand the practicalities and advantages of the proposed method.

In combination with the response of point#03 (above), the following text is available on page 20.

To evaluate the computational aspect, Table 5 indicates the computational complexity of all four DL pipelines. Undoubtedly, MoEs have nearly five times more parameters than multiclass models. This is because each MoE combines five binary experts. Comparatively, DCNN-based pipelines can be efficient at

the inference stage due to very little patch processing time per second. Similarly, MoEs have lower throughput than simpler multiclass models. As WSIs can be of different sizes, we considered reporting throughput as a more informative metric, as it scales linearly with the number of patches in a WSI. For instance, in skin cancer WSIs, MoEs roughly take 3-4 minutes per WSIs, and for urinary bladder cancer WSIs, which are 3-4 times larger, the end-to-end processing takes 10-12 minutes. We have to make a trade-off in selection, either choosing multiclass DCNN with better computational efficiency but relatively lower performance or based on the best performance. We prioritize classification performance and opt for the two best-performing DL pipelines from Table 4; therefore, we will use MoEs for the following experiments.

In addition, the results in Section 5 also highlight the performance of the different models and their computational complexity to assess their trade-offs.

5. The authors do not provide a comprehensive discussion of the potential limitations of the MoE approach, such as the increased computational complexity compared to simpler models.

In the first paragraph of the conclusion (on page 30), we mentioned, "If high inference speed is the desired requirement, then multiclass models can be a better alternative with some degree of performance trade-off." which is a conclusion derived from the results section and Tables 2, 3, and 5 in the paper.

Section 7 on page 30 discusses the limitations of the approach. The following text can be found in the manuscript.

The proposed work has a limitation in that the DL models were trained on a dataset prepared from a single cohort of data. In future work, these limitations can be overcome by pooling datasets from different cohorts in training and adopting an active learning strategy to adjust meta-learned thresholding parameters for improved sensitivity. Also, by formulating tailored fusion mechanisms for different cancer types. Furthermore, artifact-refined WSIs can be tested with the corresponding diagnostic or prognostic algorithms to assess the usefulness of artifact processing pipelines for clinical practice.

Reviewer 4

I appreciate the expanded discussion and added experiments in response to the other reviewer. My concerns have been addressed.

We want to thank the reviewer for carefully reading and appreciating the changes made to the revised version of the manuscript.
