# Supplementary material for: Equipping computational pathology systems with artifact processing pipelines: a showcase for computation and performance trade-offs
Source: BMC Med Inform Decis Mak. 2024 Oct 7;24:288. doi: 10.1186/s12911-024-02676-z (PMC11457387; doi:10.1186/s12911-024-02676-z)
Supplement: Supplementary file 1 — Supplementary Material 1: Peer review [file 12911_2024_2676_MOESM1_ESM.zip › peer review/Peer review - response letter v1.pdf]

Equipping Computational Pathology Systems with Artifact Processing Pipelines: A Showcase for Computation and Performance Trade-offs

Detailed Response to Reviewers

Authors: Neel Kanwal, Farbod Khoraminia, Umay Kiraz, Andrés Mosquera-Zamudio, Emiel A.M. Janssen, Carlos Monteagudo, Tahlita C.M. Zuiverloon, Chunming Rong, Kjersti Engan

May 3, 2024

We thank the editor and the reviewers for the constructive comments and overall positive feedback. The issues raised are listed below, followed by the corresponding responses and revisions. To facilitate reading this document, we have highlighted the feedback and concerns in red, our replies in black, and text additions in blue. Likewise, in the reviewed version of the manuscript, we have highlighted major changes made using the blue color. Language smoothing and grammatical corrections have also been done in this revision.

Reviewer 1

The authors present a very extensive effort in data collection, model development, and validation to build artifact detection models for histopathology whole slide images. Both the trained models and the dataset was made available - a welcome contribution to the community. I believe the manuscript should be accepted, although as future directions, I suggest potentially also exploring (which could perhaps be discussed):

We appreciate the reviewer for recognizing our efforts in this paper and open-science contributions to the community. We have provided our replies separately to each suggestion below.

1. How much does the artifact detection / filtering contribute to same / better downstream performance on e.g. diagnostic tasks?

We thank reviewer for highlighting the need of testing the final application of

the proposed artifact detection pipeline.

We understand that exhibiting the impact of the final application can be interesting in assessing the usability of the proposed method for clinical use cases. In the related work section (fourth paragraph on page 6), we have pointed to previous works [53, 17] that demonstrated that the presence of artifacts was detrimental to the DL model‘s accuracy. Thus, detecting histological artifacts effectively ascertains improved diagnostic performance. In other words, developing effective artifact detection methods will ultimately result in improved accuracy of diagnostic DL methods. Since we have shown experiments with three datasets, which are different cancer types with a wider range of possible diagnostic tasks, the experiments for downstream performance analysis can be completed in another (future) paper by first developing diagnostic models on the data and presenting the improvement resulting from applying the artifact processing pipeline. In addition, this experimentation is very time-consuming and can not be done during the provided revision time.

We have reported the limitation of this work in the fourth paragraph on Page 30. We have created a separate section "Limitations and Future Work" and elaborated this as our future direction.

on the second paragraph of page 30.

Furthermore, artifact-refined WSIs can be tested with the corresponding diagnostic or prognostic algorithms to assess the usefulness of artifact processing pipelines for clinical practice.

2. Can a more robustly trained model (using data from many different types of tissue) generalize directly without needing to pick a probability threshold using validation data?

We thank the reviewer for this comment and agree that annotating artifacts in different tissue types can help in creating a larger dataset and might improve model‘s ability to learn from more examples. We have highlighted the possible inclusion of diverse dataset in "Limitation and Future Work" section on page 30. However, this effort would require significant manual labor. Therefore, the benefit of including the probability threshold was to inculcate the robustness through strongly predicted patches. In other words, it improves the generalization by avoiding misclassification of unseen tissue types that are not artifacts. The results in *Table 3* signify the benefit of probability threshold over the model‘s sensitivity.

on the last paragraph of page 14,

Therefore, we introduce a probability threshold, t_s, to handle previously

unseen tissue morphology and avoid misclassifying artifact-free patches with potential diagnostic relevance.

on the last paragraph of page 30,

The proposed work has a limitation in that the DL models were trained on a dataset prepared from a single cohort of data. In future work, these limitations can be overcome by pooling datasets from different cohorts in training and adopting an active learning strategy to adjust meta-learned thresholding parameters for improved sensitivity.

3. what about penmarked regions? it is a common source of artifact in WSIs.

We thank the reviewer for the advice. Though pen markings might be present on some WSIs used for manual diagnosis, pen markings mostly give a similar visual of folded or blurry class patches as the cellular feature or tissue texture diminishes and becomes hard to observe. Since the model learns artifact-free class morphology, it is very likely to detect other artifacts that are not included as separate classes during the training. *Tables 2 and 4* show the performance of the model over artifact-free class, ascertaining that the model, in combination with the probability threshold, can find regions without artifacts.
we have added the following paragraph on page 29.

In practical scenarios, there can also be other artifacts, like pen markings, due to manual annotation of RoIs. Since the models are trained for artifact-free class, we may expect MoE to distinguish between artifact-free and other regions. It is due to the fact that pen marking gives a similar visual of folded or blurry class patches as the cellular feature or tissue texture diminishes and becomes hard to observe. Nevertheless, encountering patches with pen markings might disappear as DP workflow becomes standard in all labs, and annotation over glass slides will be done manually rather than with markers.

Reviewer 2

The presented method uses a mixture of experts (MoE) approach, where multiple deep learning models are trained to detect specific types of artifacts (e.g. blur, damaged tissue, air bubbles) and their predictions are combined to get the final artifact detection. My comments are listed below:

We thank the reviewer for the efforts in reading this work and providing valuable feedback. We have replied to each suggestion separately below.

1. More details on the annotation process and quality control of the ground truth labels could strengthen the methodology.

We agree with the reviewer that a double-check and thorough annotation will improve learning and give better quantitative results. However, annotations are usually very time-consuming, especially at 40x magnification, as used in this work. Thus, it is common to annotate at lower magnifications and mark bigger regions. We will elaborate further on the followed annotation process.

In section 3, we have clarified by adding the following at three different places on page 8 and 9.

NK received training for the task and manually annotated five artifacts (blurry areas, folded tissues, blood (hemorrhage), air bubbles, and damaged tissue). The rest of the tissue was marked as an artifact-free region. Note that not all WSIs contained five artifacts present and they were not extensively labeled as distinct tissue types since this histological data is not used for any task other than artifact detection. However, each WSI had at least one annotation for RoI or the artifact region (i.e., blur, fold, etc).......

.........FK manually annotated these WSIs for five artifacts, in a similar fashion as mentioned earlier...........

..........An expert pathologist (UK) selected and manually annotated a few WSIs based on the severity of the presence of these artifacts. Only five artifacts were carefully annotated, and the rest were marked as artifact-free regions.........

2. Evaluating the impact of the proposed artifact detection pipeline on the performance of downstream diagnostic/prognostic algorithms would provide a more complete picture.

This question was also raised by reviewer 1, question 1. Please see the answer there.

3. Authors should compare the proposed tool with current state-of-the-art at least in terms of: type of artifact detected, working magnification, quantitive/qualitative output, and other relevant aspects;

We thank the reviewer for the suggestion. We would like to point out that the area of artifact detection methods is largely unexplored, with not many papers focusing comprehensively on different artifacts, or showing qualitative and quantitative metrics. However, we have added some works focusing only on one type of artifact.

We have added a new Table (Table 3 on page 19) to provide a performance comparison of the proposed MoE against identical tasks. However, we would like to point out that, due to the lack of publicly available artifact datasets,

the existing literature uses private datasets, which cannot be used for direct comparison. Mainly because they use different data and experimental setups, which differ in various aspects.
The following Table can be found in the paper with detailed caption.

Table 3 shows validation results from other relevant works from the literature as a reference. The reported results can not be directly compared as the methods were trained using different data and varying experimental setups.

Table 3: **Comparison of the proposed mixture of experts (MoE) against the literature on identical classification tasks.** Note that the reported methods were developed using different data under different experimental setups. Thus, the results are provided for reference, not as a direct comparison. The best results are marked in bold, and the second-best results are underlined.

Task and method from literature	Accuracy (%)
Folded tissue detection by [85]	92.17
Folded tissue detection by [77]	96.7
Blur detection by [86]	93.2
Blur detection by [72]	94.4
Air bubble detection by [85]	87.33
Air bubble detection by [87]	91.5
Blood detection by [70]	85
Damaged tissue detection by [88]	90
Five artifacts - MoE-DCNNs (Ours)	<u>97.82</u>
Five artifacts - MoE-ViTs (Ours)	**97.84**

4. More discussion on limitation and future works.

In combination with the response to point 2, we have made a separate "Limitation and Future Work" section on page 30. The discussion in this section highlights the limitations of exploring the proposed MoE further for various future directions to conduct research in the artifact detection domain. To allow exploring newer approaches, we have released the dataset and code publicly. The authors will also add model weights on the Github repository upon acceptance of this paper. Our open-source contribution will pave the way for smoother development of artifact detection methods and for others to compare with our research.

The proposed work has a limitation in that the DL models were trained on a dataset prepared from a single cohort of data. In future work, we will overcome these limitations by pooling datasets from different cohorts in training and adopting an active learning strategy to adjust meta-learned thresholding parameters for improved sensitivity. Also, by formulating tailored fusion mechanisms for different cancer types. Furthermore, artifact-refined WSIs can be tested with the corresponding diagnostic or prognostic algorithms to assess the usefulness of artifact processing pipelines for clinical practice.
