# Supplementary material for: Equipping computational pathology systems with artifact processing pipelines: a showcase for computation and performance trade-offs
Source: BMC Med Inform Decis Mak. 2024 Oct 7;24:288. doi: 10.1186/s12911-024-02676-z (PMC11457387; doi:10.1186/s12911-024-02676-z)
Supplement: Supplementary file 1 — Supplementary Material 1: Peer review [file 12911_2024_2676_MOESM1_ESM.zip › peer review/Peer review fa13d21f-e0c7-4e30-b0e2-4a073a03efcb - version 1.docx]

**Reviewer reports submitted for version 1**

**Reviewer 1**

Submitted: **07 Apr 2024**

The authors present a very extensive effort in data collection, model development and validation to build artifact detection models for histopathology whole slide images. Both the trained models and the dataset was made available - a welcome contribution to the community. I believe the manuscript should accepted, although as future directions, I suggest potentially also exploring (which could perhaps be discussed): - how much does the artifact detection / filtering contribute to same / better downstream performance on e.g. diagnostic tasks? - can a more robustly trained model (using data from many different types of tissue) generalize directly without needing to pick a probability threshold using validation data? - what about penmarked regions? it is a common source of artifact in WSIs.

**Reviewer 2**

Submitted: **11 Apr 2024**

The presented method uses a mixture of experts (MoE) approach, where multiple deep learning models are trained to detect specific types of artifacts (e.g. blur, damaged tissue, air bubbles) and their predictions are combined to get the final artifact detection. My comments are listed below: 1. More details on the annotation process and quality control of the ground truth labels could strengthen the methodology; 2. Evaluating the impact of the proposed artifact detection pipeline on the performance of downstream diagnostic/prognostic algorithms would provide a more complete picture; 3. Authors should compare the proposed tool with current state-of-the arts at least in terms of: type of artifact detected, working magnification, quantitive/qualitative output, and other relevant aspects; 4. More discussion on limitation and future works
