# Supplementary material for: Equipping computational pathology systems with artifact processing pipelines: a showcase for computation and performance trade-offs
Source: BMC Med Inform Decis Mak. 2024 Oct 7;24:288. doi: 10.1186/s12911-024-02676-z (PMC11457387; doi:10.1186/s12911-024-02676-z)
Supplement: Supplementary file 1 — Supplementary Material 1: Peer review [file 12911_2024_2676_MOESM1_ESM.zip › peer review/Peer review fa13d21f-e0c7-4e30-b0e2-4a073a03efcb - version 2.docx]

**Reviewer reports submitted for version 2**

**Reviewer 1**

Submitted: **12 May 2024**

I appreciate the expanded discussion and added experiments in response to the other reviewer. My concerns have been addressed.

**Reviewer 2**

Submitted: **10 May 2024**

This paper proposes a DL approach for detecting artifacts in WSIs to improve the reliability of computational pathology systems. My major concerns are listed as follows: - The paper does not compare the proposed approach with existing state-of-the-art methods for artifact detection. The authors should provide a comprehensive comparison with relevant methods from the literature. In the last few years, several methods (most of them open-source) to evaluate artefact in digital pathology (es. HistoQC); - While the authors create a pipeline that provides quantitative data, there is no numerical control or validation of the errors. For example, the authors should provide an absolute error comparison between manually and automatically estimated artifact areas to demonstrate the accuracy of their approach. - The paper lacks a discussion on important practical considerations, such as computation times, compatibility with different formats and scanners, and the effect of magnification on the proposed approach. - The computational time and resources required for training and inference are not reported. This information is essential for readers to assess the practicality and feasibility of the proposed approach, especially when considering the trade-offs between performance and computational complexity. - The authors do not provide a comprehensive discussion of the potential limitations of the MoE approach, such as the increased computational complexity compared to simpler models.
